# Plant Pest Detection Using an Artificial Nose System: A Review

**DOI:** 10.3390/s18020378

**Published:** 2018-01-28

**Authors:** Shaoqing Cui, Peter Ling, Heping Zhu, Harold M. Keener

**Affiliations:** 1 Department of Food, Agricultural and Biological Engineering, The Ohio State University/Ohio Agricultural Research and Development Center, 1680 Madison Ave, Wooster, OH 44691-4096, USA; cui.411@osu.edu (S.C.); keener.3@osu.edu (H.M.K.); 2 United States Department of Agriculture-Agricultural Research Service (USDA-ARS) Application Technology Research Unit, 1680 Madison Ave, Wooster, OH 44691-4096, USA; heping.zhu@ars.usda.gov

**Keywords:** electronic nose, pest scouting, pest management, gas sensor, noninvasive detection

## Abstract

This paper reviews artificial intelligent noses (or electronic noses) as a fast and noninvasive approach for the diagnosis of insects and diseases that attack vegetables and fruit trees. The particular focus is on bacterial, fungal, and viral infections, and insect damage. Volatile organic compounds (VOCs) emitted from plants, which provide functional information about the plant’s growth, defense, and health status, allow for the possibility of using noninvasive detection to monitor plants status. Electronic noses are comprised of a sensor array, signal conditioning circuit, and pattern recognition algorithms. Compared with traditional gas chromatography–mass spectrometry (GC-MS) techniques, electronic noses are noninvasive and can be a rapid, cost-effective option for several applications. However, using electronic noses for plant pest diagnosis is still in its early stages, and there are challenges regarding sensor performance, sampling and detection in open areas, and scaling up measurements. This review paper introduces each element of electronic nose systems, especially commonly used sensors and pattern recognition methods, along with their advantages and limitations. It includes a comprehensive comparison and summary of applications, possible challenges, and potential improvements of electronic nose systems for different plant pest diagnoses.

## 1. Introduction

Reliable disease and pest diagnosis in the early stages of vegetable and fruit production is highly desirable to reduce major production and economic losses. The main purpose of plant pest diagnosis is to assess whether a plant is healthy and to determine the causes of a disorder, if any. However, one major challenge is the difficulty in determining the physical, chemical, and biological changes in plants during the asymptomatic stages of an infection. Another challenge lies in the difficulty of performing the task timely and economically. 

To address these challenges, diverse methods or technologies have been developed, which can be divided into two methods: direct and indirect. Direct detection methods include molecular technologies, including polymerase chain reaction (PCR), fluorescence in-situ hybridization (FISH) and serological technologies such as enzyme-linked immunosorbent assay (ELISA) [1,2,3]. Meanwhile, typical indirect methods detect morphological changes, transpiration rate changes and volatile organic compounds (VOCs) profiles, which correspond to the technologies of fluorescence imaging, hyperspectral techniques and gas chromatography–mass spectrometry (GC-MS) [4,5,6]. Besides, specific biosensors, such as antibody-based biosensors, DNA/RNA-based affinity biosensors and enzymatic electrochemical biosensors have been developed based on bio-recognition. DNA-based and serological methods are the most available and essential direct detection tools for accurate plant disease diagnosis, providing “standard methods” for fungal detection [7]. However, as discussed in other review papers, they are not very reliable at early (asymptomatic) stages and require at least 1–2 days for sample harvest, processing and analysis [8,9]. Thus, there is a need for rapid, reliable diagnostic methods that can be used in the field for crop disease detection at asymptomatic stages. Indirect methods that rely on imaging techniques and VOCs profiles released from infested crops have the potential to address this need. For example, hyperspectral image techniques utilized in both field and greenhouse grown plants for early detection of stress have shown satisfactory classification accuracy. However, some modifications and improvements, such as instantaneous results, are still needed [7]. Biosensors using phage display and bio-photonics have been reported to instantaneously detect infections but still require modifications, improvements and proper validation before being used in the field. In the past three years, the principles behind these technologies, as well as their advantages and disadvantages, have been thoroughly discussed in several review papers and numerous original research papers; thus, this review emphasizes novel sensors and techniques based on VOCs profiles, which are promising technologies for reliable, non-destructive and real-time plant disease monitoring and management [10,11,12]. The advantages and disadvantages of the aforementioned technologies are discussed in Table 1 [13,14,15,16,17,18,19,20,21,22,23,24,25,26].

Detection of plant infections prior to the onset of visual symptoms is valuable for executing appropriate management strategies and pest control to prevent the spread of diseases [27,28]. Plants emit a large amount of VOCs, which deliver functional information related to their growth, health, and disease [29]. 

Plants have a broad range of defense mechanisms for combatting infections, attacks by herbivorous insects, and mechanical damage [30]. One of these protection strategies involves emitting specific VOCs to fight potential attackers. These defenses are often divided into direct defenses and indirect defenses. In direct defenses, plants emit repellent VOCs to reduce insect attacks, while, in indirect defenses, VOCs can attract predators to battle pests. It is clear that VOCs play significant roles in plant communication and present promising functionality for improving crop protection [31]. VOCs emitted from plants indicate their real-time physiological health status and could provide bio-information that could be used for rapid, non-invasive disease diagnosis. Moreover, the composition of VOCs varies according to the type of damage, such as pathogen infection and herbivore feeding [32]. Some VOCs are present as a strong aromatic gas, but most of them occur at extremely low concentrations that are below the human olfactory threshold. Therefore, novel sensors, sensing technologies, and data analysis methods are required to detect plant VOCs and to interpret the information. The development of such technologies is critical for utilizing VOCs profiling concepts in the field for improved crop production. 

GC-MS is a conventional technique used for separating and identifying individual VOCs [33,34]. However, GC-MS is expensive and time-consuming; it does not function in real time; and it requires specific expertise for compound determination. Thus, electronic noses (E-noses) are being evaluated to detect plant VOCs [35,36]. This paper provides a comprehensive review of this novel technology, including sensor arrays, sampling set design, pattern recognition, and challenges. Applications and potential improvements for using E-noses to diagnose pest-infected plants are also discussed.

## 2. Electronic Nose Detecting Technology

E-noses, also known as artificial olfaction devices, have been widely developed over the past two decades. They have been extensively employed in diverse applications ranging from medical diagnosis to the food industry, environmental protection, and agriculture [37,38,39,40]. These systems are designed to mimic the mammalian olfactory system. They are coupled with different types of sensor arrays, which transform the VOCs information into an electronic signal. When gas samples are spread across the sensor array, the odor molecules induce reversible physicochemical changes to the sensing materials. This causes changes in electrical properties such as the resistance and electrical potential. Conditioning circuits are used to modulate the signals, and pattern recognition is used to classify the aromas. Finally, the data can be read, displayed, and saved for pattern recognition analysis, which could provide basic diagnosis results. The most important parts of E-nose system, gas sensors and pattern recognitions, are briefly discussed below. 

### 2.1. Gas Sensors 

Several commercial gas sensors are available for E-nose systems. They can be grouped according to working mechanisms into three different categories: conductivity sensors, gravimetric sensors, and optical sensors. 

#### 2.1.1. Conductivity Sensors 

Conductivity sensors are based on a conducting polymer (CP) and/or metal oxide semiconductor (MOS), both of which work on the principle of variations in conductivity or resistance upon exposure to particular gases. Although the response mechanisms are different, the physical structures, such as sensing materials, electrodes, and substrates of the conductive sensors, are basically the same. MOS-based sensors need an extra heater. 

Conducting polymers have many advantages over other materials when used as gas sensors. Sensors prepared from conducting polymers can operate at room temperature. This is a critical advantage for portable battery-powered E-nose systems since a heater significantly increases power consumption, reducing battery life. More importantly, high discrimination in array sensors can be achieved by using different conducting materials due to the various categories of conducting polymers that are available. However, a main drawback of conducting polymer composites is aging, which can cause sensor drift and poor performance. Furthermore, these materials are not sensitive to certain gases. For example, a sensor based on a graphene oxide-based composite is not sensitive to trimethylamine (TMA), which is a typical VOC released from decaying fish. 

In recent years, due to their simple fabrication and variety, conductive polymer-based sensors have been used in detecting wood decay. The AromaScan 32S is a commercial E-nose with 32 organic conductive polymer-based sensors that have been used to determine incipient wood decay caused by fungi. After pre-training on the aroma of pure fungus cultures as well as healthy and decayed wood samples, unknown samples of wood decay fungus were correctly identified based on their VOCs, with up to 93.2% accuracy [41]. Fast sensor response and good repeatability were also demonstrated in the detection process. Conducting polymers-based sensors, however, have a short life-time, and humidity can affect sensor performance. 

MOS sensors are some of the most commonly used gas sensors for constructing sensor arrays due to their cost-effectiveness, reliability, and availability. They have been widely applied in agriculture and forestry industries for diagnosis of plant infection caused by fungus, bacteria, and viruses; insect damage; or mechanical damage [42,43,44,45,46,47]. The main advantages of MOS sensors are the fast response and recovery times, which mainly depend on the temperatures and the level of interaction between the sensors and gases [48]. MOS sensors are small and can be constructed as integrated circuits. However, the applications of MOS sensors are limited to “moderate” gases such as CO_2_ and H_2_, and they are not suitable for sulfur containing gases which can bind with the sensing materials [48,49,50,51]. The operation of MOS sensors requires high temperatures of around 200–500 °C, which is beyond the temperature range that a common battery can achieve and, thus, limits practical field applications. 

#### 2.1.2. Gravimetric Sensors

Two types of gravimetric sensors are employed in E-nose systems: surface acoustic wave (SAW) sensors and quartz crystal microbalance (QCM) sensors. SAW sensors produce a surface wave that travels along the surface of the sensor, while QCM sensors produce a wave that travels through the bulk of the sensor. The working principle of both sensors involves a change in the mass of the piezoelectric sensor coating due to gas absorption, which results in a change in the resonant frequency upon exposure to VOCs [52]. 

A SAW sensor consists of a piezoelectric substrate with an interdigital input receiving electrode and output transmitting electrode located on the top surface of the substrate. A sensitive thin film is located between the interdigital electrodes. Odor molecules interact with the sensing film and change the mass of the entire sensor unit which leads to a change in the frequency. 

**Figure 1:**
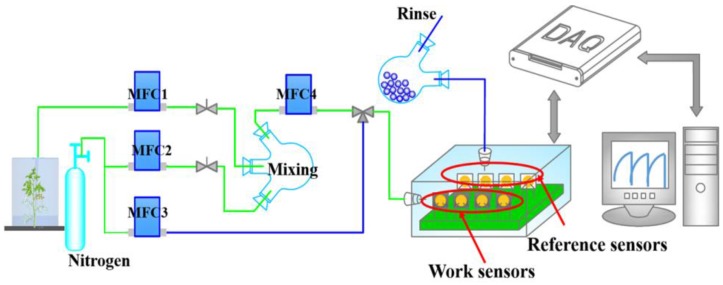
An E-nose system based on QCM sensor array. MFC, mass flow control; DAQ, data acquisition.

QCM-based sensors have a similar operating principle to SAW sensors but a different device structure. The sensor is composed of a quartz chip coated with an absorbing sensing membrane, and a set of gold electrodes attached to the bottom of the chip, with one on each side. Cui et al. investigated the feasibility of an E-nose based on QCM sensors for predicting the shelf life of fruits and meats. The sensor showed promising performance for evaluating food quality [53,54]. The sensitivity and selectivity of these sensors strongly depend on the type of sensing material and the interaction between the odor and film compounds. Improving the sensitivity of such sensors relies on developing specific sensing materials for specific VOCs biomarkers. The author’s research group developed an ultra-sensitive E-nose system, consisting of a QCM sensor array with four conducting polymers (Figure 1), specifically for infested plants. The primary results indicated that this system has the potential to provide an accurate diagnosis in real-time. The advantages of using SAW and QCM sensors include low cost, small size and high sensitivity. However, they have some disadvantages, such as a complex fabrication process and circuitry and a short life span [55]. 

#### 2.1.3. Optical Sensors

In contrast to the aforementioned sensors, the mechanism of optical sensors is based on changes in chemical properties, such as the reactivity, redox potential, and acid-base interactions [56]. Optical sensors use a wavelength-selectable light source, a light detector, and sensor materials that interact with gases. Colorimetry and fluorometry are the two typical techniques used for analyzing the signal obtained from optical sensors. Suslick and Rakow developed the first colorimetric sensor array in a cartridge package for use in odorant detection [57]. A difference map can be obtained from a digital image by digital subtraction of the image of the array before and after exposure. The advantages of colorimetric sensors are their disposability, fast response, and strong robustness for hazardous gas detection. However, a major drawback for many optical sensors is unexpected sensitivity to humidity in the environment, especially for real-time detection, since the humidity varies from day to day and from indoors to outdoors. Research showed that these optical sensors respond to humidity with relative humidity concentrations ranging 10–95% [58,59,60]. A significant change in signal caused by a change in humidity can cover up the signals of the target odorants. This issue can be addressed by using hydrophobic materials as substrates for colorimetric or fluorometric sensor arrays. However, a drawback of colorimetric optical sensors is their short lifetime, since molecular dyes used as sensing materials have a limited shelf life [61]. Due to their high sensitivity (sub-ppb), optical sensors and metric arrays have been widely used in the detection of toxic industrial chemicals, explosives, foods and beverages, bacteria, and cancer [62,63,64]. The advantages and disadvantages of the aforementioned sensors are summarized in Table 2.

### 2.2. Sampling Methods

#### 2.2.1. Laboratory Sampling

**Figure 2:**
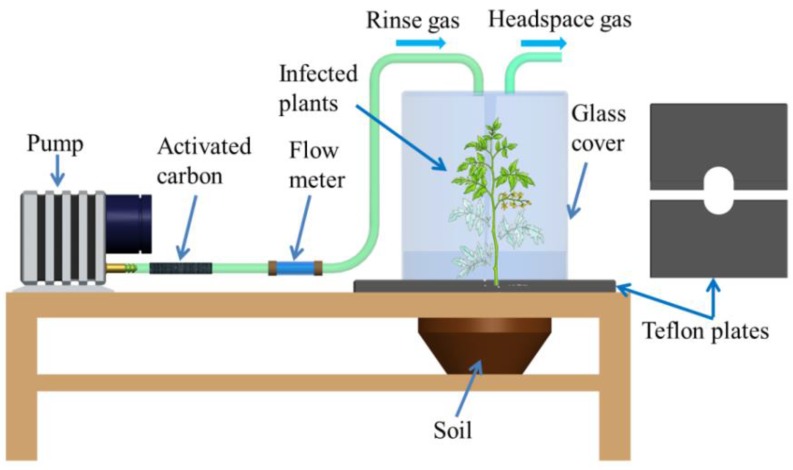
Illustration of VOC collection system for infected plants.

Odor compounds are drawn into an E-nose via different collection methods, such as headspace sampling, diffusion methods, bubblers, and pre-concentrators. Profiling plant VOCs are conventionally carried out in a sealed chamber or box with controlled temperature and humidity, which simulate the environment of a greenhouse and field. A typical sampling setup is shown in Figure 2. In one study, rice plants with different pest damage were placed in the container, and VOCs were collected after 20 min to allow for static headspace build up before sampling [46]. In another study, an E-nose was employed to sample the VOCs emitted by powdery mildew and spider mite infected tomato plants, which were housed in clear glass boxes. The humidity and temperature were logged at all times. During the cultivation, clean air was pumped in to create positive pressure to maintain constant environmental parameters and decrease the risk of cross contamination [69,70].

#### 2.2.2. Field Sampling

The continuous changes in VOCs, temperatures, and humidity in open space conditions have impeded large scale field applications of E-nose. Recent studies have attempted to address these challenges. One potential solution is cultivating plants in a field environmental control chamber under natural light. In one reported attempt, two-year-old potted apple and pear plants were enclosed in plastic bags or Teflon chambers and cultured under field conditions in a shelter for E-nose detection. The temperature and CO_2_ assimilation were controlled to maintain consistent levels [71]. Another detection experiment was performed directly at the site of basal stem rot in infected oil palm plants and the surrounding soils using a portable commercial E-nose (Cyranose 320). The results showed ~99% accuracy in identifying infected trunks and soils from healthy ones [72]. Biondi et al. explored the feasibility of using E-noses for detecting brown rot and ring rot in potatoes in laboratory and field conditions. Results indicated that the E-nose was able to distinguish between healthy potato samples and infected ones under the designed conditions, which included storage in polypropylene bags in a refrigerated chamber [73]. Most of these attempts required an enclosed space and climate control capability to keep the environment relatively stable. A feasible, low cost and easy to use method for field sampling could be gas collection directly from plants or plant branches covered with polyethylene terephthalate bags to form a relatively stable environment. However, the concentrations of VOCs were found to be relatively low for this method, but could be pre-concentrated by extending the sampling time from 30 min to 3–6 h. The detection of VOCs from plants in open fields or greenhouses is still a big challenge. 

### 2.3. Data Analysis Methods

**Figure 3:**
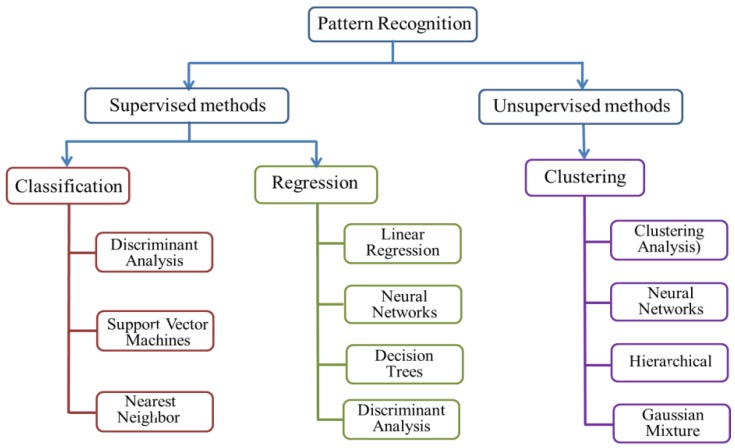
Typical pattern recognition methods applied on E-nose system.

Data analyses using algorithms are used to perform qualitative classifications and quantitative predictions. There have been significant improvements in pattern recognition technologies, and many advanced algorithms have been introduced for E-nose systems. Two classes of statistical methods are generally used, as shown in Figure 3 The first, supervised methods, include artificial neutral networks (ANN), and are used to classify unknown features of a class that have the most common properties based on prior knowledge or probability distributions from training samples [74]. The other group is unsupervised methods, such as cluster analysis (CA), which separate the input data into different clusters based on feature similarity [75]. To provide a general overview for applications in plant health determination, the sections below review the four most common approaches: cluster analysis (CA), ANN and random forest (RF). 

#### 2.3.1. Unsupervised Statistical Methods

CA is a widely applied unsupervised classification technique in which clusters are determined based on the distance between each data point [76]. The most common clustering algorithm is Ward’s minimum variance method, which minimizes the total data within the cluster variance. The resultant dendrogram shows the connectivity and distance between each of the clusters, in which the shorter the distance, the more similar the samples. A CA dendrogram provides a straightforward way of displaying cluster similarity with semi-quantitative results. Laothaworbkitkul et al. employed an E-nose and cluster analysis to distinguish VOCs emitted from control, artificially damaged, herbivore-damaged, and diseased plants (cucumber, pepper, and tomato plants) [72]. The results of CA clearly showed clusters between damaged and undamaged plants. As expected, CA can also successfully differentiate cucumbers infested with spider mites from wounded cucumbers and healthy cucumbers [77]. The advantages of CA include revealing associations and structures in data which were not previously evident and presenting results in an easy to understand dengrogram. However, some methods are still not clearly established and there is no complete satisfactory method for determining the appropriate number of clusters.

#### 2.3.2. Supervised Statistical Methods

ANNs are supervised learning algorithms and are best known for their good adaptability properties in learning, generalization, and noise tolerance, making them suitable for processing nonlinear data. ANNs are capable of learning from input data and optimizing neuron weights in real-time through iterative training and self-adjustment. ANNs consist of multiple layers of neurons, which depend on the complexity of the system. The outputs of ANNs depend on the design of the experiment. Due to their robustness and self-adaptability, ANNs have been introduced to E-nose systems to play the role of a “brain” and provide accurate quantitative analysis. Shakaff et al. employed an E-nose combined with an ANN as the main pattern recognition method to detect oil palm trees infested with basal stem rot disease. After training with 240 samples, a typical three-layer network, with one input layer, one hidden layer, and one output layer, was established to analyze another 160 samples. The classification was 100% successful when using the multilayer perceptron and probabilistic neutral network algorithms, while a 97.5% success rate was achieved when using the radial basis functions (RBF) algorithm. All three of these methods are ANN methods but use different types of supervision [78]. A back-propagation feed-forward artificial neutral network (BP-ANN) has also been employed to differentiate different apple cultivars, and showed a satisfactory accuracy of 87% [79]. Compared with unsupervised methods, supervised methods such as ANN require a large amount of training samples, but they provide more robust algorithms and higher accuracy. Besides, ANNs require less formal statistical restrictions on the input variables and they are able to learn and model complex nonlinear relationships between dependent and independent variables. However, the trained “black box” tends to be over-fitted due to the empirical nature of model development.

As the most popular supervised learning algorithm, RF is an ensemble learning method for both classification and regression, which has been widely used as a classifier and predictor in analyzing E-nose data. Briefly, RF is a combination of tree (decision) predictors. The value of a random vector decides a single tree predictor individually and for all the others trees [80]. Its proceeds are generally operated by constructing a multitude of decision trees at training time and providing the class that is the mode of the classification or averaged prediction of the individual trees. Specifically, bootstrap sampling is firstly established and subsets are generated based on bootstrap sampling distributions and randomly original dataset with replacement. For each data subset, a corresponding decision tree model is built. The above three steps are repeated until all the tree models are grown. Finally, the class membership of new samples will be predicted by a maximum vote of the predictions [81]. Due to its good performance in both classification and regression, RF has been intensively adopted in E-nose data analysis. A RF classifier was established to discriminate the difference between healthy maize plants and those at an early stage of *Phaeosphaeria* leaf spot infestation. Results showed an overall accuracy of 88% and a kappa value of 0.75, indicating that RF has potential as a classifier in detecting maize disease infestation [82]. A model to predict powdery mildew infection levels of chardonnay grape brunches was reported to achieve an accuracy of 0.87 in classification of healthy, infected and severely diseased bunches [83]. It is obvious that RF is efficient for a large database and could give an estimate of the important variable in the classification, but it also tends to be over-fitted for some datasets with noisy classification and regression tasks. The advantages and disadvantages of the aforementioned pattern recognition methods were summarized in Table 3. 

## 3. Applications in Plant Diagnosis

**Figure 4:**
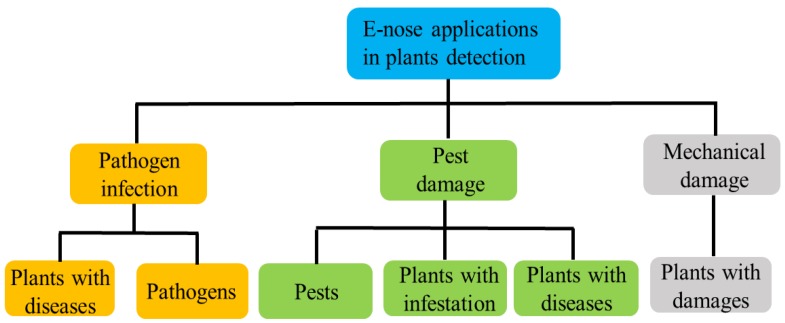
The applications of E-nose in plants disease detection.

Plant VOCs play significant roles in responding to pest attacks and are promising targets for pest detection. The composition of VOCs emitted by plants depends on the mode of damage. Biologically, VOCs are produced by a wide range of physiological processes in many different parts of plant tissues. The defense mechanisms to pathogens or viruses are still unclear, but the VOCs have been found to change after plants are infected. Plants require a broad range of defense mechanisms to effectively combat attacks by herbivorous insects or mechanical damage [30]. One of the strategies is to emit specific VOCs to battle potential attacks. While some protective VOCs are emitted at all times, others are induced only in response to herbivore feeding [84]. Accordingly, theses variations of VOCs provide reliable principles of pest detection via E-nose, as shown in Figure 4. Discussed below are advances of using E-noses in detecting fungal and bacterial infections and insect infestations in plants. The discrimination of damaged plants caused by mechanical damage is also discussed, as it is considered as background noise for VOCs detection of insect damage. 

### 3.1. Fungal and Bacterial Disease Infections

E-noses have considerable potential in detecting plants with fungal or bacterial infections. The direct approach involves detecting VOCs released from isolated microbes using an E-nose, while the indirect method involves determining changes in VOCs emitted from infected plants that have been inoculated with a target fungus or bacteria.

There has been success using E-noses for early diagnosis to discriminate fire blight (*Erwinia amylovor*) and blossom blight (*Pseudmonas syringgae* PV. Syringae) on apple trees under controlled laboratory conditions [85] and field conditions [71]. Researchers have also demonstrated that fire-blight-infected pear trees can be successfully detected in the early stages of infection using an E-nose based on MOS sensors. Grapevines inoculated with tumorigenic strains of *Agrobacterium vitis* have been correctly differentiated from healthy groups with 83.3% accuracy using a portable E-nose system [71]. Tomato plants, one of the most valuable greenhouse crops in the world, have attracted the most interest among researchers for investigating VOC fingerprints to monitor the plant’s health status. Zhang et al. explored the effects of powdery mildew on the VOCs of infected tomato plants under greenhouse conditions. The results indicated that the disease had a major effect on the VOC profile, and the E-nose was able to discriminate between infected plants and healthy ones with classification accuracy of over 94% [69,70]. Levels of infection by *Ceratocystis fagacearum* (oak wilt) were predicted with 78.65% accuracy using E-nose detection [86]. 

Infection microbes (fungi, bacteria, and viruses) might also contribute to the VOC profile of infected plants. Therefore, it is also essential to detect volatile compounds released from microbial metabolites or during microbial culture. The fire blight pathogen (*E. amylovora*) was found to have unique volatile characteristics and has been differentiated from other plant-associated bacteria using a MOS-based E-nose, achieving 87.5% accuracy in discrimination from a reference species [43]. A satisfactory distinction between *E. coli* and Listeria from individual colonies of suspension was accomplished with 92.4% classification accuracy [87]. VOCs of bacterial strains directly isolated from both chilies and papaya plants were measured and analyzed using an E-nose system, and further confirmed the feasibility of E-nose diagnosis of pathogenic bacteria in plants. 

There is also good potential for detecting asymptomatic diseased plants infected with various plant pathogens using E-noses. However, most experiments and validations have been carried out in lab conditions. In field conditions, limitations should be considered and improvements are still needed. For example, plants are usually infected with several diseases during growth and concentrations of volatiles are under the threshold of many commercially available sensors. The unique VOCs associated with a target disease may also be covered or diluted by background VOCs, which are inconsistent in open areas. Therefore, the technology could be greatly improved by developing sensitive and selective sensors; determining specific VOC biomarkers for distinct plant diseases; and validating E-noses or sensor arrays in nurseries, greenhouses, and field conditions. 

### 3.2. Insect Damage 

E-noses have been successfully demonstrated for detecting insect infested plants and insect population dynamics. Spider mites (*Tetranychus urticae* Koch) are herbivorous arachnids that can feed on several hundred host plants, including economically important tomato plants cultured in greenhouses. E-noses have been used for extensive investigations of VOCs profiles emitted by tomato plants infested with spider mites, under different growth conditions [69,70]. Tomato plants can be correctly classified, without a priori knowledge, as healthy or infected using this technique. 

The reliability of E-nose technology has been confirmed in rice plant diagnosis. Infected rice plants attacked by the striped rice stem borer (*Chilo suppressalis*) and the brown planthopper (*Nilaparvata lugens*) can be easily discriminated from healthy plants. Furthermore, the extent of damage to the rice plant as well as the amount of pests can also be successfully estimated based on the sensor response of an E-nose [88]. Volatiles released from insects such as brown planthoppers and stink bugs might be another avenue for revealing different aspects of insect-infested plants. Xu et al. demonstrated the possibility of estimating the age and number of brown planthoppers using an E-nose with classification accuracies of 100% and 48.93%, respectively [89]. Mating disruption, which involves attracting or confusing males to impede mating and reduce the number of insects, has been widely adopted. Therefore, early recognition of insect gender in a rapid and practical way is critical for applying mating disruption. The gender and species of stink bugs have been precisely predicted using a portable E-nose [45,46]. Although the preliminary studies and results discussed thus far show that E-noses with an appropriate detection methodology are promising, the development of intelligent E-noses for specific insect detection is urgently required. 

### 3.3. Mechanical Damage

Besides pathogenic and insect damage, mechanical damage also arouses defense responses and causes changes to VOCs fingerprints. The composition of VOCs emitted by damaged plants may depend on the mode of attack [69]. Zhou and Wang investigated differences in the VOCs patterns of rice plants under pest attack and mechanical damage using an MOS-based E-nose. The results indicated that VOCs caused by the mechanical damage were different from those caused by pest attacks, with a classification accuracy of 91.9% using principle component analysis [46]. Although mechanical damage does not account for a major loss of crop yield, there are plenty of crops that could be damaged by mechanical equipment used in the field. More importantly, the VOCs information from pest damage can be easily confounded by that from mechanical damage. Therefore, detection of VOCs from mechanical damage is recommended during the determination of pest damage.

## 4. Challenges and Improvements 

Extensive studies have demonstrated in laboratory environments that E-noses are promising non-destructive tools for quick and early plant pest damage detection. For field applications, however, several areas of improvement are necessary. 

### 4.1. Dynamic Nature of VOCs

VOCs emissions of plants are specific to different tissues, locations, mass, and physiological stages. α-thujene, a pheromone in aphids (*Homoptera*) and allomone in termites (*Isoptera*), has been reported to accumulate in peduncles of pistachio trees (*Pistacia vera*), with rarely any amount in the leaves and fruits [90]. The VOCs profile is also dynamic throughout the plant’s life cycle. For example, the composition of a flower’s odor profiles and the total odor production reach maximum levels when the flower is ready for pollination. After pollination, the amount of VOCs starts to decrease until the end of the flower’s life cycle [90,91,92,93]. Seasonal variations and location are other factors that significantly affect the VOCs profile. Son et al. investigated the effect of seasonal change on the emission fluxes of monoterpene released from coniferous trees. They found that the amounts of monoterpenes emitted from pitch pine and Korean pine were generally highest in spring, followed by summer and fall, while they were lowest in winter [94]. The VOCs fingerprinting of the same type of American ginseng cultivated in two different locations showed significant differences, indicating that origin strongly influences the VOCs fingerprints [95,96]. Therefore, the dynamic nature of VOCs profiles due to differences in region, age, season, and tissues increases complexity in characterizing VOCs biomarkers for the task of pest detection. This is a major challenge for disease diagnosis, even for the same species of plants. 

### 4.2. Environmental Effects on Sensing

The influence of humidity and temperature is another challenge in the detection of plant pests, as sensors in E-nose systems are sensitive to these factors. The instability of these two factors causes obvious drift in the sensor response, which reduces the signal to noise ratio (S/N) for detection of the targeted VOCs. Furthermore, humidity reduces the lifetime of gas sensors, thus limiting their application in long-term and high frequency monitoring of a plants’ status. Preconditioning the sample gases and housing the sensors in a dry environment can mitigate the aforementioned limitations; however, the power requirements of these units may constrain their field applications. 

### 4.3. Detection in Field Conditions

The performance of E-noses in actual production environments needs to be studied using larger field trials. However, the environmental parameters of open fields, such as temperature, humidity, and background gas compositions [97,98], are uncontrollable and keep changing. Furthermore, the concentrations of many VOCs released from plants are very low and below the detection threshold of currently available E-noses. Background noise generated from the atmosphere can also hide the plants’ true VOCs variance caused by pest attacks, fungal infection, or other causes. Therefore, a controlled environment that can maintain temperature, humidity, and even gas compositions, to certain degree, is more suitable for E-nose applications. Further development of sensor arrays with high sensitivity and selectivity are desirable. 

### 4.4. Plant Pest Specific Detection Technique Optimization

As previously discussed, the artifact of natural variations of plant released VOCs profiles might overshadow the true changes caused by the presence of targeted pests. Moreover, the characteristic VOCs that reflect a specific pest infection of plants remain unknown. Thus, specific VOCs closely related to a plant pest might be ignored or mistakenly identified; therefore, the identification of distinct volatile biomarkers specific to a particular pest or plant is required, and sensitive and selective gas sensors that are specific to these biomarkers need to be optimized. Attempts to address this challenge should firstly determine the characteristic volatile biomarkers through a combination of conventional precision chromatography technologies, such as GC-MS. The next step is to fabricate the corresponding sensing materials that exhibit high sensitivity and selectivity to the target volatile indexes, and finally to develop a special gas sensor array based on these sensing materials. 

### 4.5. Combinations with Other Advanced Technologies

It is well known that E-noses are designed to identify the entire fingerprint of VOCs, but not individual volatile components. However, information about specific components is essential when detecting hazardous or toxic gases. Therefore, the advantages of conventional chromatography technologies are obvious. The combination of an E-nose system with mass spectrometry or gas chromatography could address these challenges and extend the application of these technologies. Inspired by comprehensive sensory evaluation of biological senses, an electronic tongue that mimics the human sense of taste could be combined with an E-nose to establish a more robust and more widely applicable platform, especially for applications requiring liquid detection or in areas with high humidity [87,88]. 

### 4.6. Micro E-Noses 

An inexpensive portable E-nose would be preferable for plant pest detection, especially in open field detection. However, the bulky size and high price of most commercially available E-noses limit their potential agricultural applications. One improvement would be to develop a small (one-chip) or micro-level sensor array by using integrated circuit (IC) technologies and micro-electro mechanical systems (MEMS) to reduce the size [99]. Moreover, the price of ICs can be relatively low with mass production. Therefore, a new generation of portable E-noses with extremely small size and low cost could be realized. 

With the rapid development of smart phones, a one-chip sensory array could be integrated with smart phones and a user-friendly interface in an app, which could help to realize intelligent, small, multi-functional, and low-cost E-noses. In this scenario, a smart phone with an E-nose chip could diagnose plant pests by placing it near infected plants, and the results could be displayed immediately. Identifying and quantifying VOCs emitted from plants can enhance E-nose sensors to maximize their effectiveness. Further refinements, such as the design and optimization of sensor arrays for specific VOCs markers, are likely to lead to improvements in sensitivity, as well as increase the robustness of the technology in the face of inconsistent environments in production agriculture. 

A miniaturization of hardware units, such as signal conditioning and data acquisition components, is another way to reduce the size of E-nose system. The potential advantages of such a system include low cost, small size and wide application due to portability. Extensive research has been done to design portable E-nose systems by adopting a micro-controller equipped with a compact flash memory that assures data acquisition, analysis in real-time, and light-emitting diode (LED) screen [100,101]. Those designed portable E-nose systems have been successfully used for food inspection and indoor air quality monitoring [102]. 

## 5. Conclusions 

Plant pests threaten commercial crops, causing reduction of food production and leading to significant economic losses worldwide. To control and manage the damage caused by pests, various technologies and different strategies have been developed and used. Although conventional methods, such as nucleic acid and serology-based technologies, have been commercially available and widely used, their applications for field detection is limited due to the need of a laboratory setting, time consumption for analysis, and cost limitations. The current and newly developed technologies, such as imaging methods or biosensors, have attracted extensive attention but still need improvement. Therefore, developing advanced, real-time, cost efficient and portable devices or technologies for early stage detection are needed. A portable E-nose system equipped with sensitive gas sensor arrays and pattern recognition is an innovative method that may meet these requirements, as well as provide some advantages over traditional technologies and address some challenges such as field-application. 

An overview of innovative E-nose technologies with an emphasis on applications in plant pest detection were reviewed in this paper. The major advantages of E-noses include being extremely sensitive, providing real-time analysis, and being easy to operate and portable; thus, E-nose technology can provide a new platform for plant pest diagnosis. It has been demonstrated, in both laboratory and field environments, that plant infection symptoms can be successfully diagnosed using E-noses with accurate prediction and satisfactory sensor performance. However, challenges remain in regard to sensor selectivity, interference from the surrounding atmosphere, and the difficulty of detection in open fields, which require further investigation and improvement. 

## Figures and Tables

**Table 1:** Comparison of the typical technologies for plant disease detection.

Techniques	Advantages	Disadvantages	Refs.
PCR	Mature technology, easy to operate and portable	Subjected to DNA extraction, and inhibitors and polymerase activity	[13,14]
FISH	Highly sensitive	Auto-fluorescence	[15,16,17]
ELISH	Low-cost, rapid and visible results	Low-sensitivity to bacteria	[18]
Fluorescence imaging	Sensitive to abnormalities in photosynthesis	Limited in field setting	[19,20]
Hyperspectral Techniques	Rapid and highly robust	Affected by external factors, such as light, view angle; relatively expensive	[21]
GC-MS	Providing individual VOCs information	Expensive, not real-time, expertise skills needed	[22,23]
Enzymatic biosensor	Real-time and high specificity	Unstable, easily affected by pH, environment	[24]
DNA-based biosensor	Low cost, low limit of detection	Easily affected by DAN extraction, not real-time	[25]
Antibody-based biosensor	Low cost	Not real-time	[26]

**Table 2:** Summary of advantages and disadvantages of gas sensors applied on E-noses [65,66,67,68].

Name	Advantage	Disadvantage
CP ^1^	Wide range of available conducting polymers; room temperature operation; fast response; sensitive to polar compounds	High sensitivity to humidity and temperature; sensor response drift with time; short-life time
MOS ^2^	Small size; easy to integrate into measurement circuitry; fast response and recovery time; high sensitivity	High-power-consumption; limited application on portable systems; blind with sulfur gas; limited coating materials; sensitive to humidity
SAW ^3^	Broad applications; high sensitivity; fast response; diverse sensing materials; small size;	Relatively poor signal to noise performance; complex circuitry; unsatisfactory reproducibility
QCM ^4^	Fast response time; easier fabrication compared to SAW; high sensitivity; diverse sensing materials; small	Unsatisfactory reproducibility; complex circuitry
CM ^5^	High sensitivity; fast response; robustness in hazardous environment; disposable after use	Sensitive to humidity; complex supporting software and instrument; short life time; only sensitive to oxygen and VOCs

^1^ CP, conducting polymer; ^2^ MOS, Metal Oxides Semi-conducting; ^3^ SAW, Surface Acoustic Wave; ^4^ QCM, Quartz Crystal Microbalance; ^5^ CM, Colorimetric.

**Table 3:** Summary of advantages and disadvantages of CA, ANN and RF.

Name	Functions	Advantages	Disadvantages
CA	Classification	Reveal associations and structures in data which are not evident; results are easy to understand	Some methods are not clearly established; no satisfactory method for determining the appropriate number of clusters
ANN	Classification, regression and prediction	Require less formal statistical restrictions; able to model complex nonlinear relationships; able to train multiple algorithms	Big computation burden; tend to overfit
RF	Classification, regression and prediction	Efficient for large database; estimate the important variable in the classification; generate forests for further use	Overfitting for some datasets with noisy classification and regression tasks
